# Supplementary figures and images for: Role of the novel aloe vera-based titanium dioxide bleaching gel on the strength and mineral content of the human tooth enamel with respect to age
Source: PeerJ. 2024 Sep 18;12:e17779. doi: 10.7717/peerj.17779 (PMC11416088; doi:10.7717/peerj.17779)

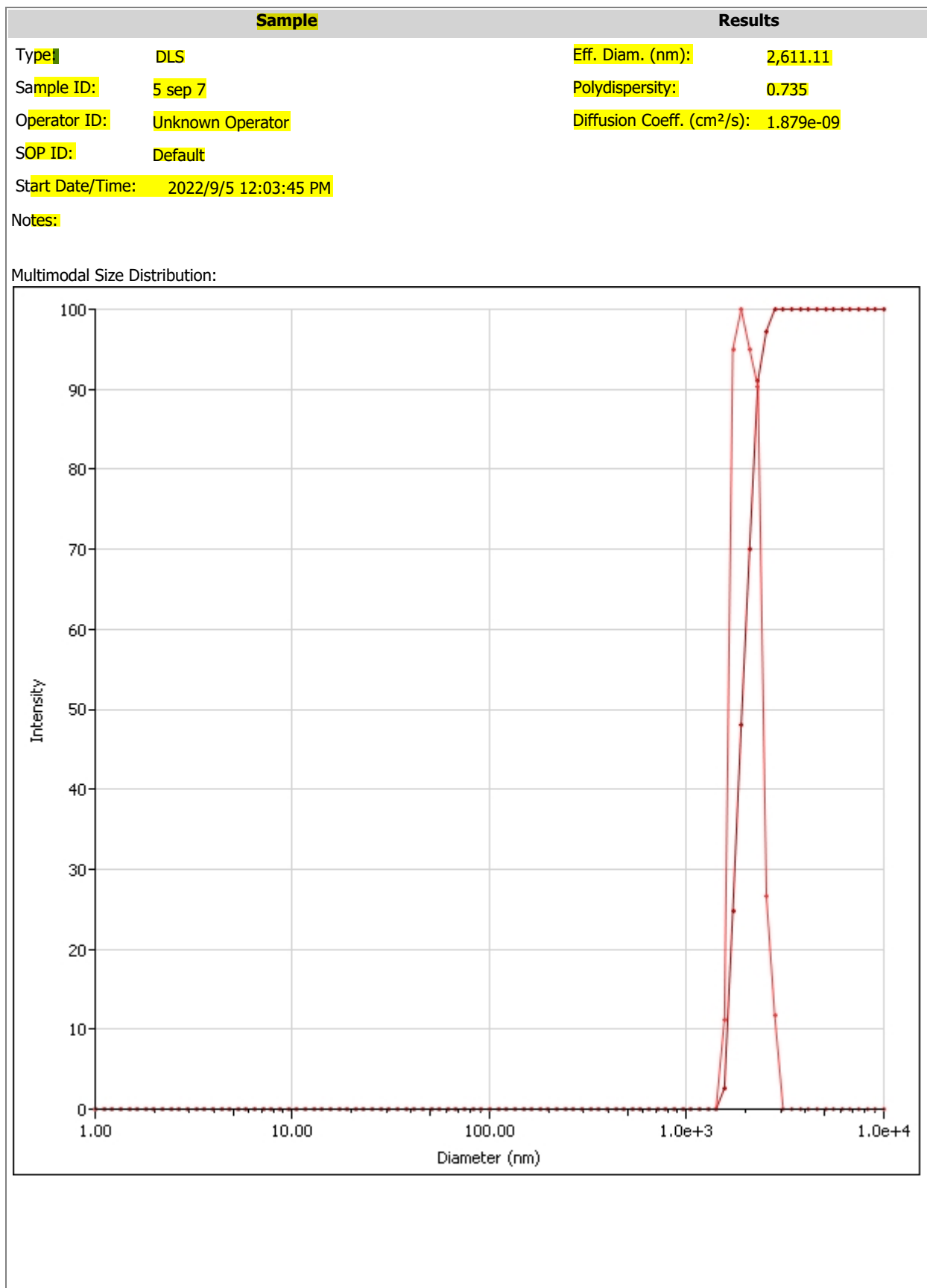

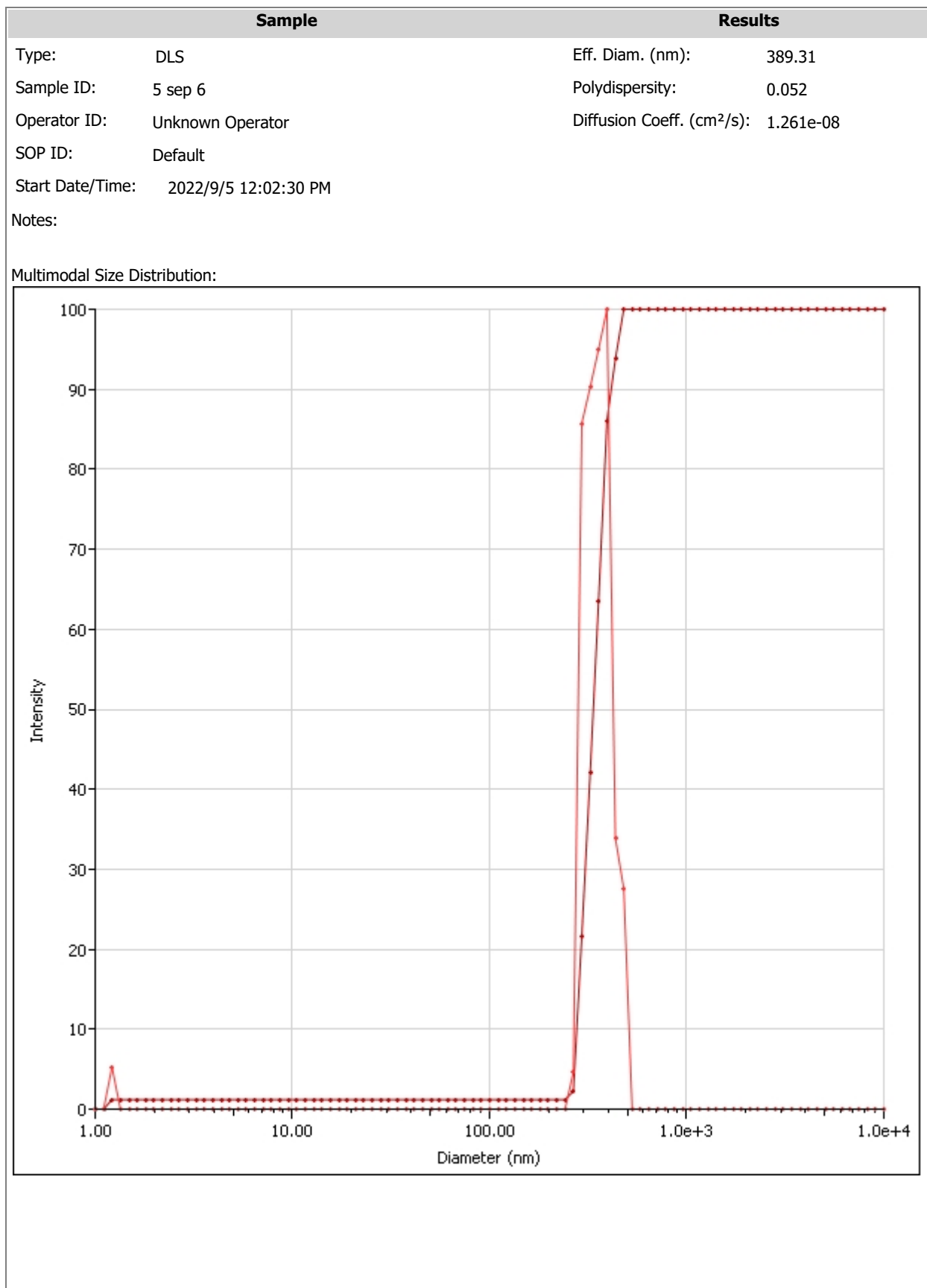

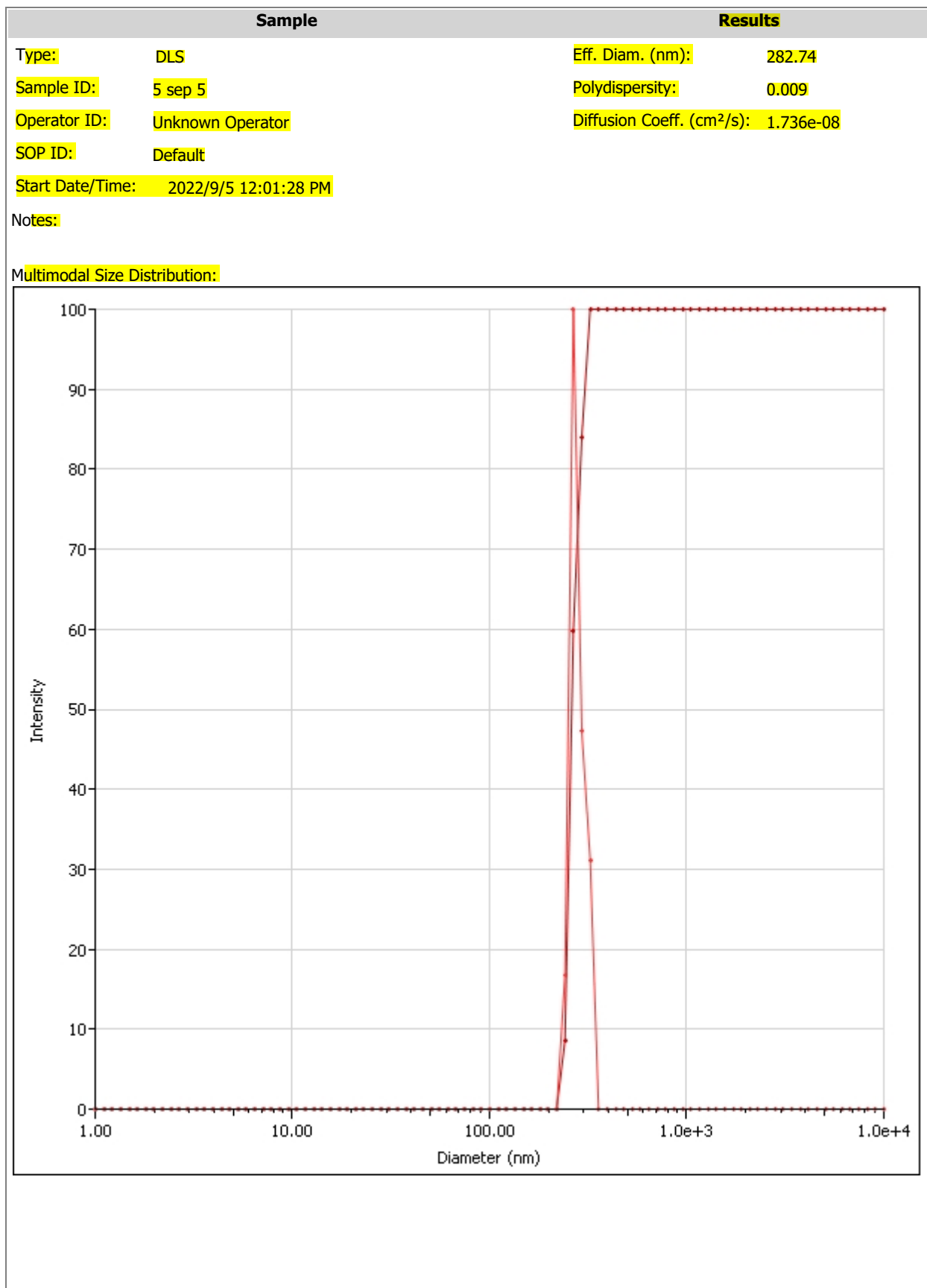

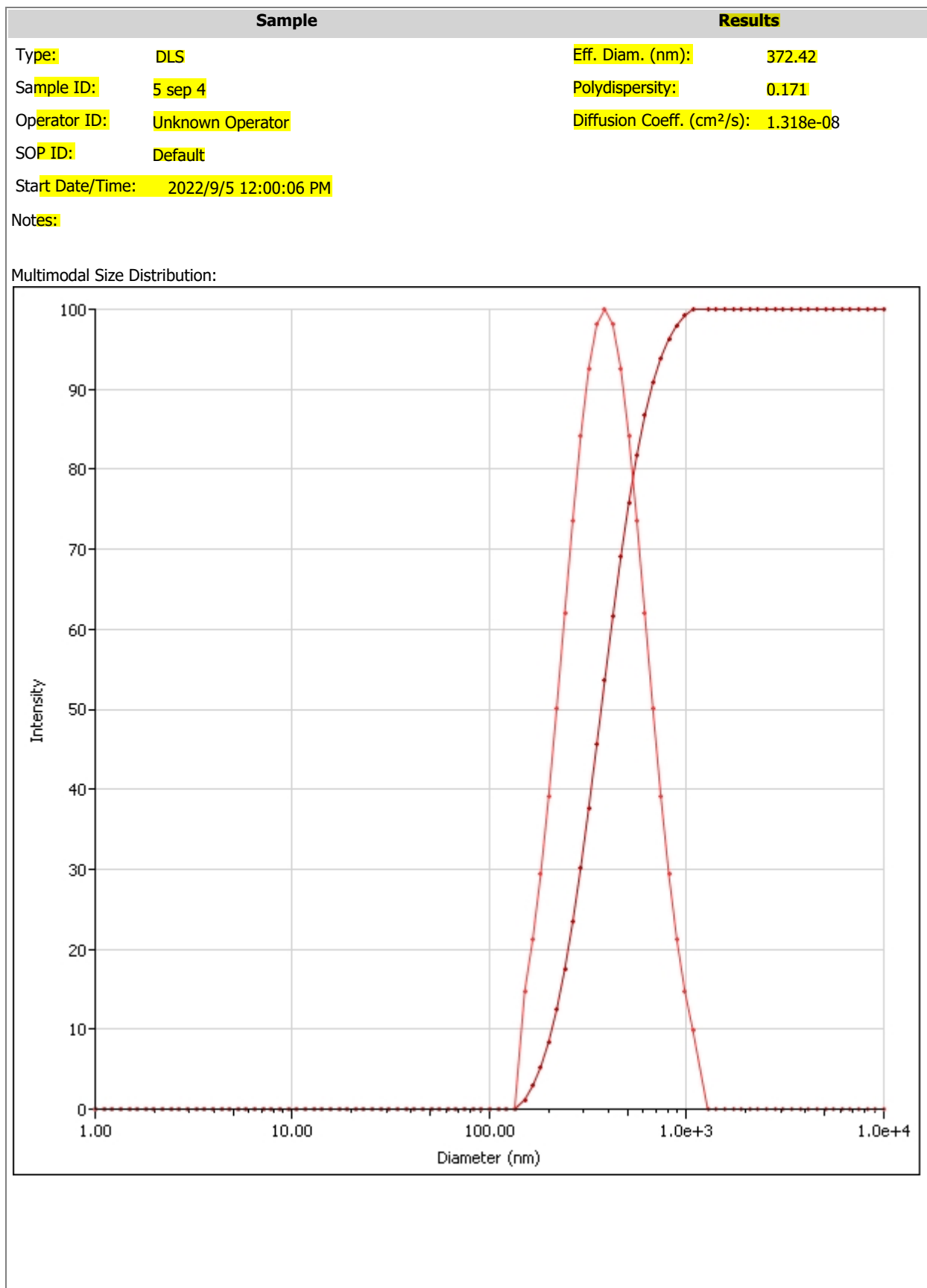

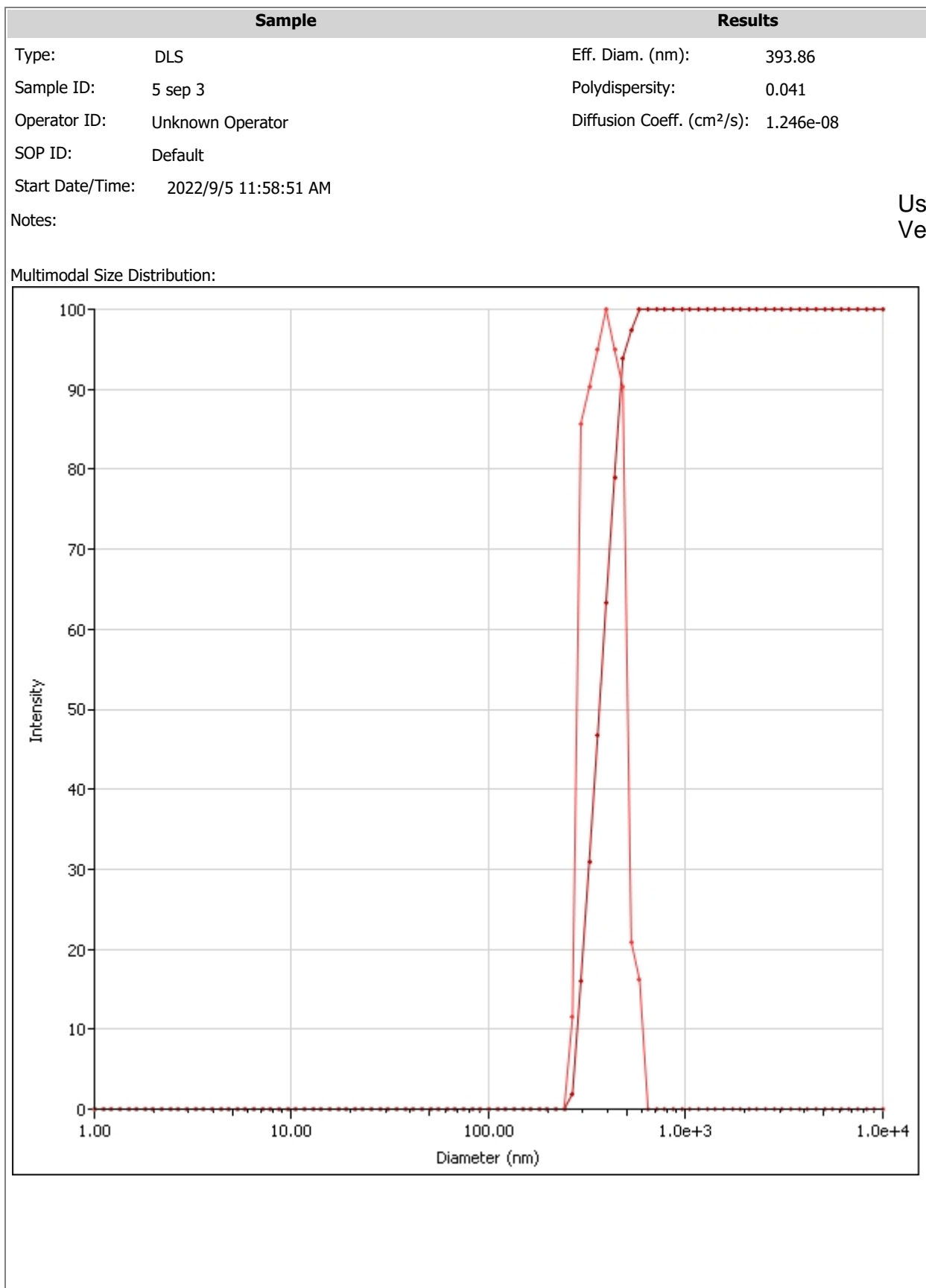

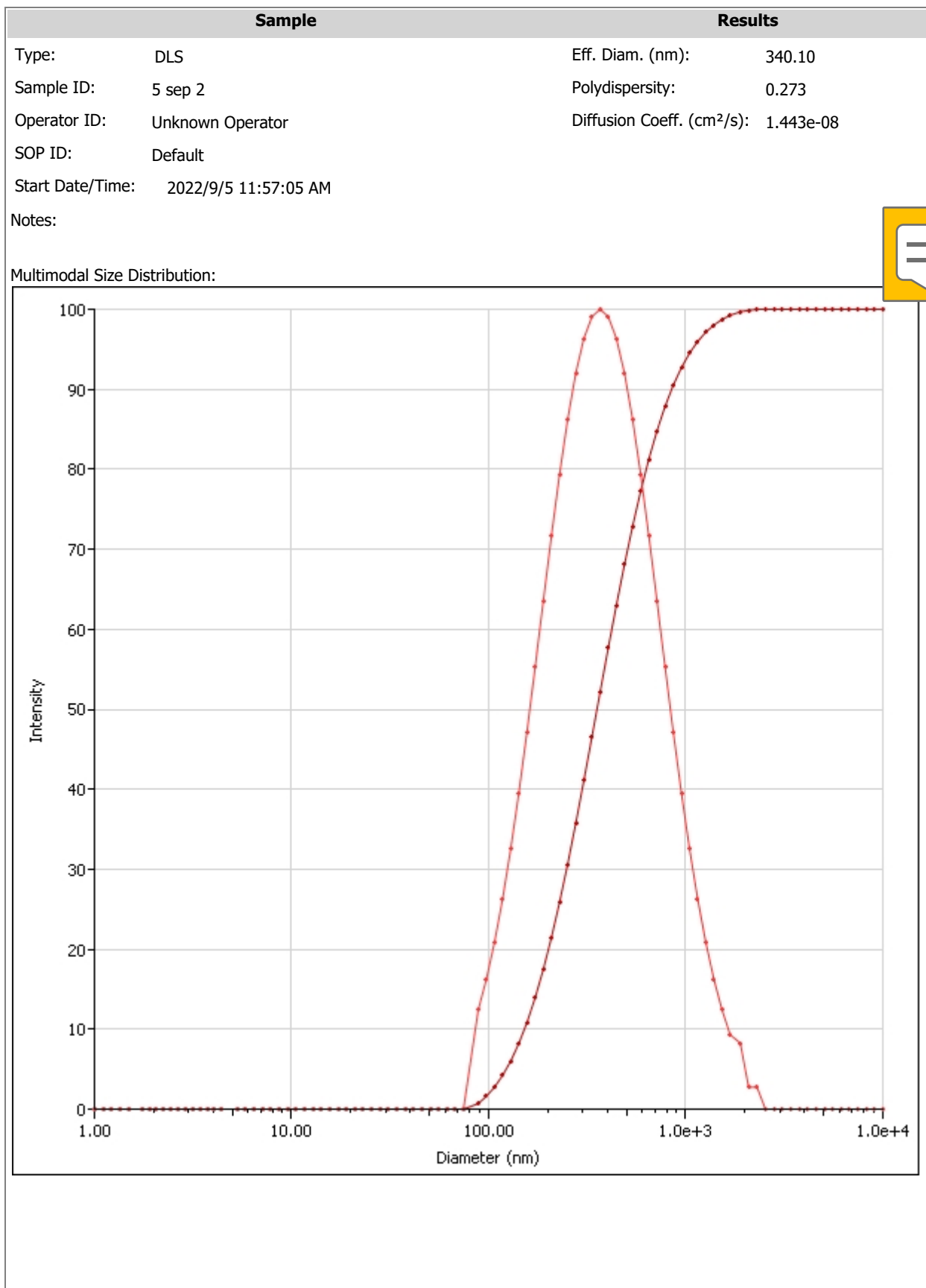

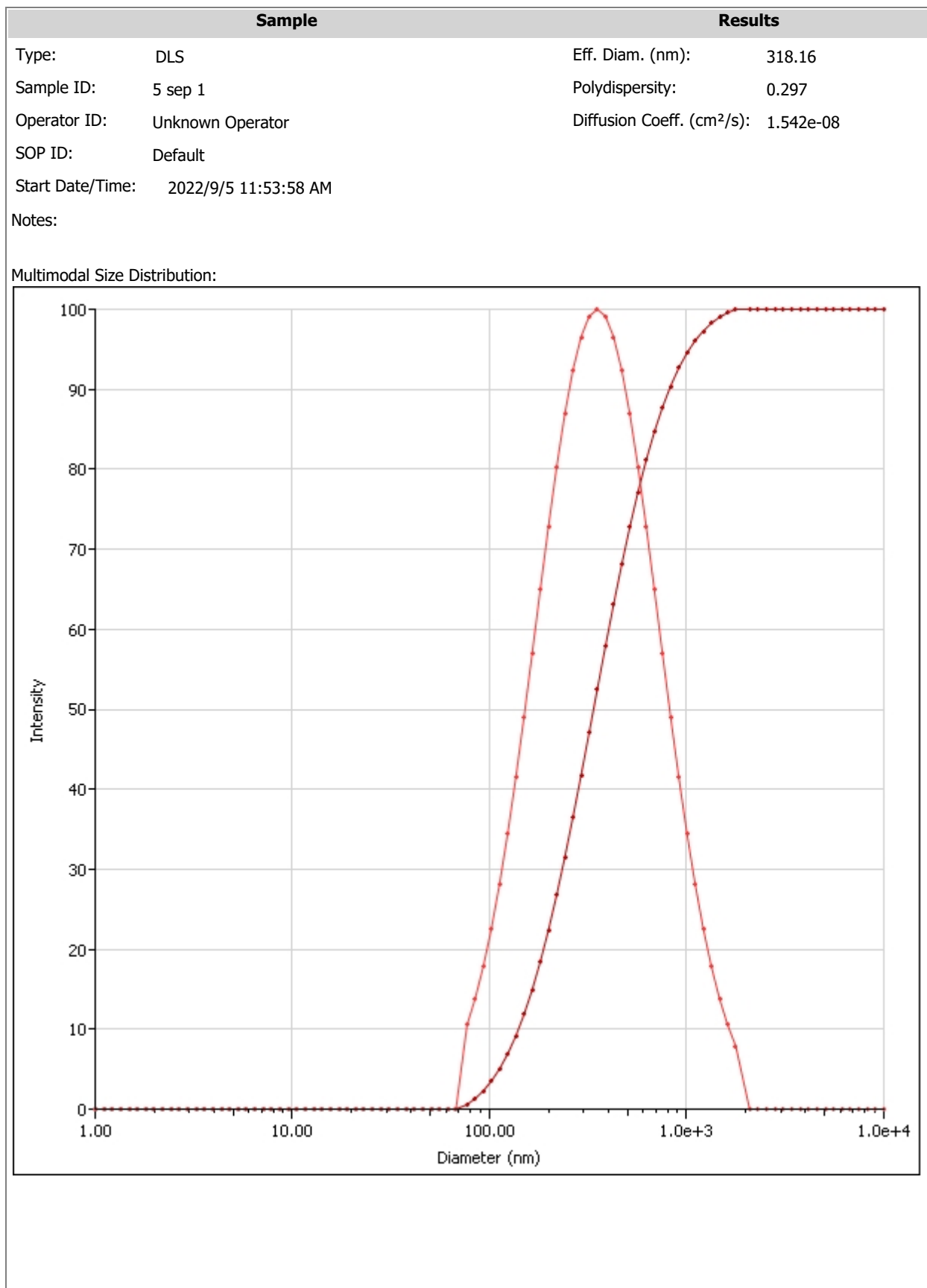

Supplement: Supplemental Information 8 [file peerj-12-17779-s008.pdf]
