# Supplementary material for: Role of the novel aloe vera-based titanium dioxide bleaching gel on the strength and mineral content of the human tooth enamel with respect to age
Source: PeerJ. 2024 Sep 18;12:e17779. doi: 10.7717/peerj.17779 (PMC11416088; doi:10.7717/peerj.17779)
Supplement: Supplemental Information 9 [file peerj-12-17779-s009.pdf]

Dr. Afsheen

Author: ISRC
Creation: 3/10/2021

Sample Name: 2

Area 1

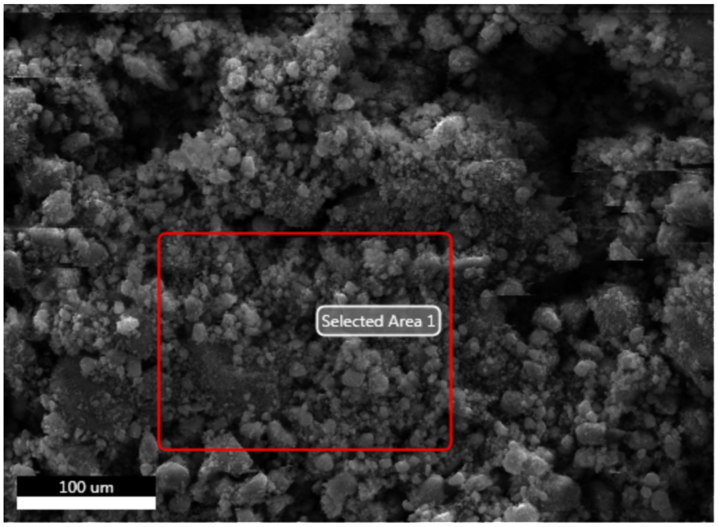

Notes:

Selected Area 1

kV: 30	Mag: 250	Takeoff: 18.6	Live Time(s): 30	Amp Time(µs): 7.68	Resolution:(eV) 123.9

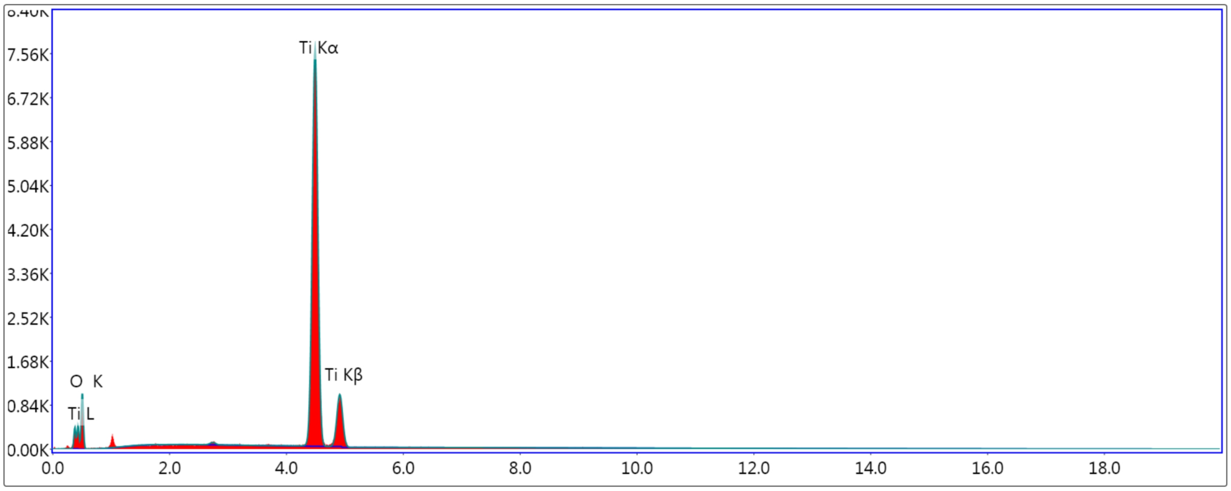

Lsec: 30.0 0 Cnts 0.000 keV Det: Octane Pro Det Reso

eZAF Smart Quant Results

Element	Weight %	Atomic %	Net Int.	Error %
O K	47.29	72.87	158.33	11.31
TiK	52.71	27.13	2938	1.27
